# Supplementary material for: A Method to Monitor the NAD+ Metabolome—From Mechanistic to Clinical Applications
Source: Int J Mol Sci. 2021 Sep 30;22(19):10598. doi: 10.3390/ijms221910598 (PMC8508997; doi:10.3390/ijms221910598)
Supplement: Supplementary file 1 [file ijms-22-10598-s001.zip › ijms-1376881 supplementary conversion corrected.pdf]

Supplementary Materials

A Method to Monitor the NAD^+ Metabolome—From Mechanistic to Clinical Applications

Maria Pilar Giner [1], Stefan Christen [1], Simona Bartova [1], Mikhail V. Makarov [2,3], Marie E. Migaud [2], Carles Canto [1] and Sofia Moco [1,4,*]

[1] Nestle Research, EPFL Innovation Park, H, 1015 Lausanne, Switzerland; mariapilar.giner@rd.nestle.com (M.P.G.); stefan.christen@rd.nestle.com (S.C.); simona.bartova@rd.nestle.com (S.B.); carles.cantoalvarez@rd.nestle.com (C.C.)
[2] Mitchell Cancer Institute, University of South Alabama, 1660 Springhill Avenue, Mobile, 36604, Alabama, USA; mmigaud@southalabama.edu
[3] Olon Ricerca Bioscience, 7528 Auburn Road, Concord, 44077, Ohio, USA; mikhail.makarov@olonricerca.com
[4] Division of Molecular and Computational Toxicology, Department of Chemistry and Pharmaceutical Sciences, Amsterdam Institute for Molecular and Life Sciences, Vrije Universiteit Amsterdam, De Boelelaan 1108, 1081 HZ Amsterdam, The Netherlands
* Correspondence: s.moco@vu.nl

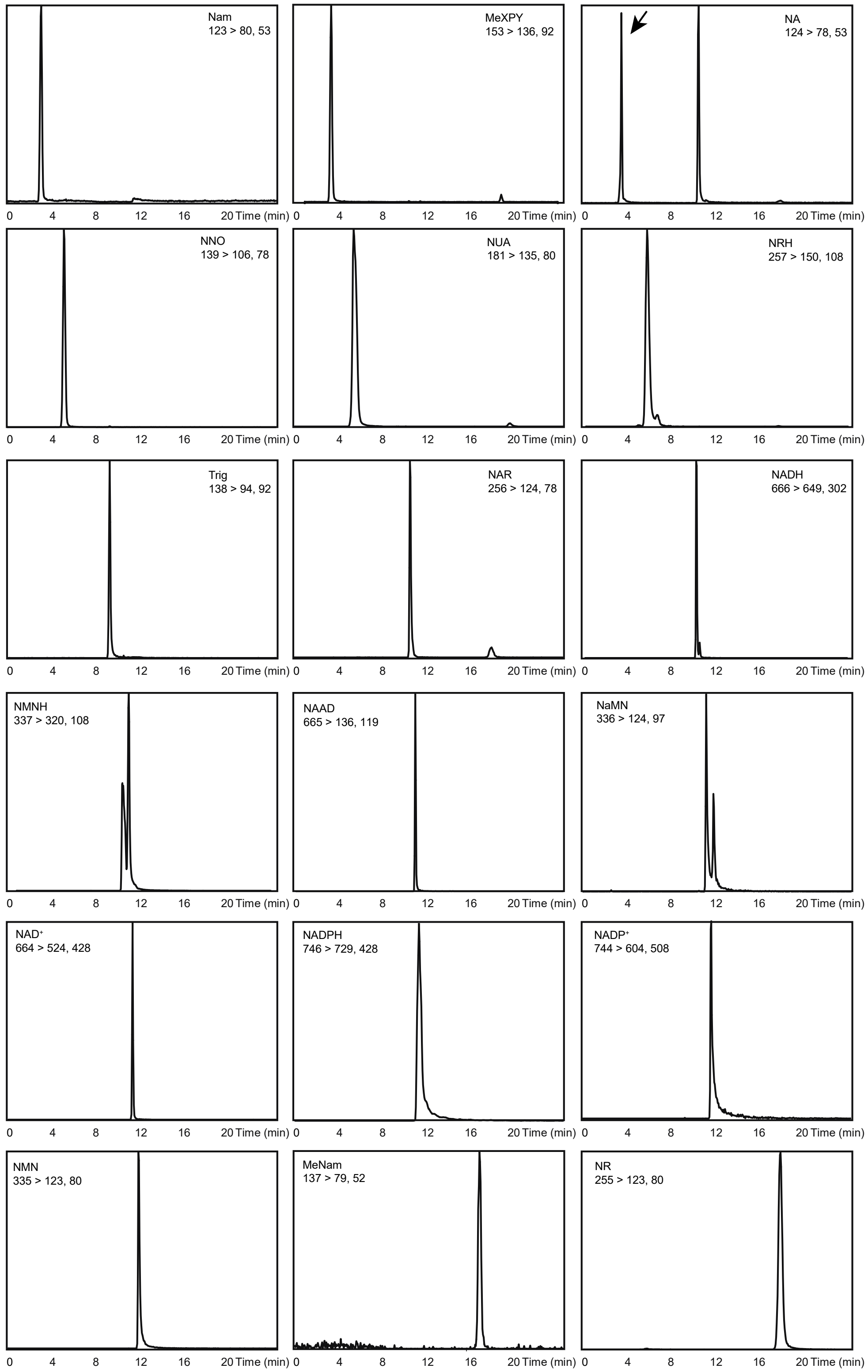

Figure S1. HILIC-ESI$^+$-MS/MS chromatograms of the 18 metabolites part of the NAD$^+$ metabolome (standard compounds) used in this study, obtained from the analysis of pools of standard compounds solutions, expressed in relative abundance (arbitrary units), and maximized per analyte. The arrow indicates the signal of NA (that has a chemical interference with NAR at a later retention time).

Table S1. Comparison of 3 sample preparation procedures in detecting the NAD+ metabolome of Hep G2 cells: biphasic extraction (BE), 80% cold methanol (80MeOH) and 40:40:20 with 0.1 M formic acid (40:40:20) extraction (n = 5, average ± SD). Values expressed in ratios of LC-MS signal intensity normalized to internal standard and total protein content (arbitrary units).

Extraction	Nam	NRH	Trig	NADH	NMN	NR	NMNH	NAD+	NADPH	NADP+
					**Average**					
BE	0.25	0.01	0.01	6.85	0.07	0.14	0.00	11.02	2.66	0.67
80MeOH	0.15	0.02	0.01	6.17	0.06	0.10	0.01	10.37	1.40	0.63
40:40:20	0.10	0.01	0.00	4.64	0.05	0.00	0.01	9.44	2.11	0.59
					**Standard Deviation**					
BE	0.04	0.00	0.00	0.91	0.01	0.03	0.00	0.68	0.19	0.09
80MeOH	0.02	0.00	0.01	0.65	0.01	0.01	0.01	0.91	0.23	0.12
40:40:20	0.02	0.01	0.00	0.11	0.00	0.00	0.01	0.83	0.22	0.04

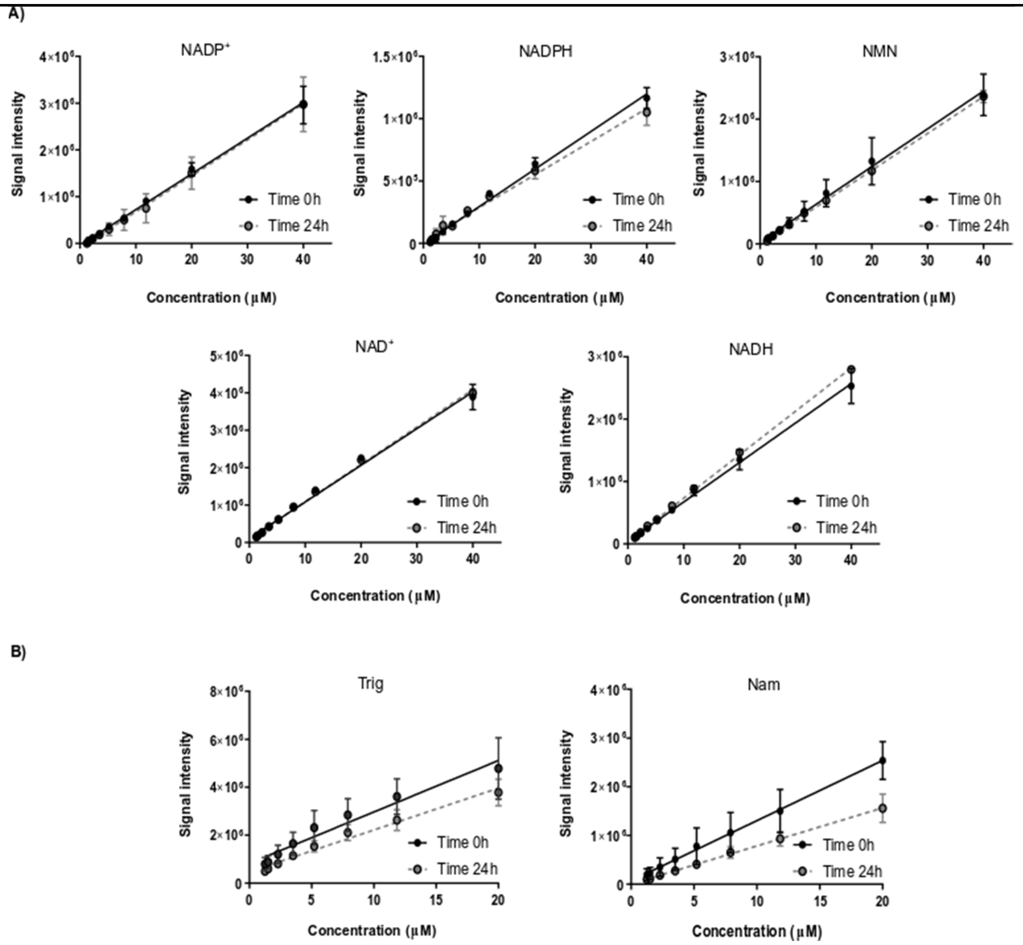

Figure S2. LC-MS metabolite signal intensity for NAD+ metabolites in standard solutions before and after 24 h at 4 °C (autosampler temperature) for a concentration range **A,** 1.25–40 µM (9 points) or **B,** 1.25–20 µM (8 points). Average ±-standard deviation values (n = 3).

Table S2. NAD^+ metabolome of Hep G2 cells upon administration of Nam, NR or NRH (0.5 mM) and control (Ctrl), (n = 4, average ± SD). Values expressed in ratios of LC-MS signal intensity normalized to internal standard and total protein content (arbitrary units).

	Nam	NRH	NADH	NMN	NR	NMNH	NAD^+	NADPH	$NADP^+$
**Average**									
Ctrl	0.52	0.04	2.84	0.06	0.29	0.00	6.39	1.52	0.43
NA	0.38	0.02	3.32	0.06	0.21	0.00	6.51	1.46	0.50
Nam	5.58	0.01	3.14	0.06	0.21	0.01	7.24	1.47	0.48
NR	3.21	0.63	3.17	0.16	99.74	0.01	9.92	1.50	0.58
NRH	1.11	100.60	19.17	4.60	148.27	17.21	98.01	2.37	0.72
**Standard Deviation**									
Ctrl	0.19	0.01	0.49	0.01	0.10	0.01	1.22	0.26	0.09
NA	0.06	0.01	0.55	0.01	0.08	0.01	0.35	0.16	0.06
Nam	0.58	0.00	0.15	0.01	0.08	0.01	0.41	0.22	0.06
NR	0.97	0.15	0.75	0.04	24.44	0.02	0.87	0.20	0.05
NRH	0.13	11.10	1.24	0.16	25.77	1.06	2.25	0.33	0.03

Table S3. NAD^+ metabolome of murine plasma, urine, kidney, liver, pancreas, and muscle.

ID	Urine (n = 8)	Plasma (n = 7)	Kidney (n = 5)	Liver (n = 8)	Pancreas (n = 7)	Muscle (n = 8)
Nam	X	X	X	X	X	X
MeXPY	X	X	X	X	X	X
NA	-	-	-	-	-	-
NNO	X	X	-	-	-	-
NUA	-	-	-	-	-	-
NRH	X	X	X	X	X	X
Trig	X	X	X	X	X	X
NAR	-	-	-	-	-	-
NADH	-	-	X	X	X	X
NMNH	-	-	-	-	-	-
NAAD	-	-	-	-	-	-
NAMN	-	-	-	X	-	-
NAD^+	-	-	X	X	X	X
NADPH	-	-	X	X	X	X
$NADP^+$	-	-	X	X	X	X
NMN	X	-	X	X	X	X
MeNam	X	-	X	-	-	-
NR	X	X	X	X	X	-

ID: metabolite acronym; X = detected metabolite; - = not detected metabolite; n = number of biological replicates tested.

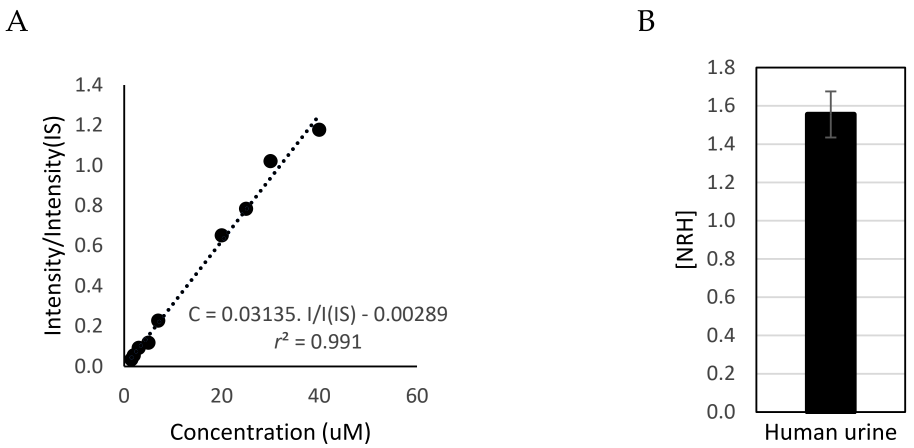

Figure S3. (**A**) Calibration curve of NRH; (**B**) quantification of NRH (μM) in 2 urine replicates (pool of at least 2 donors).

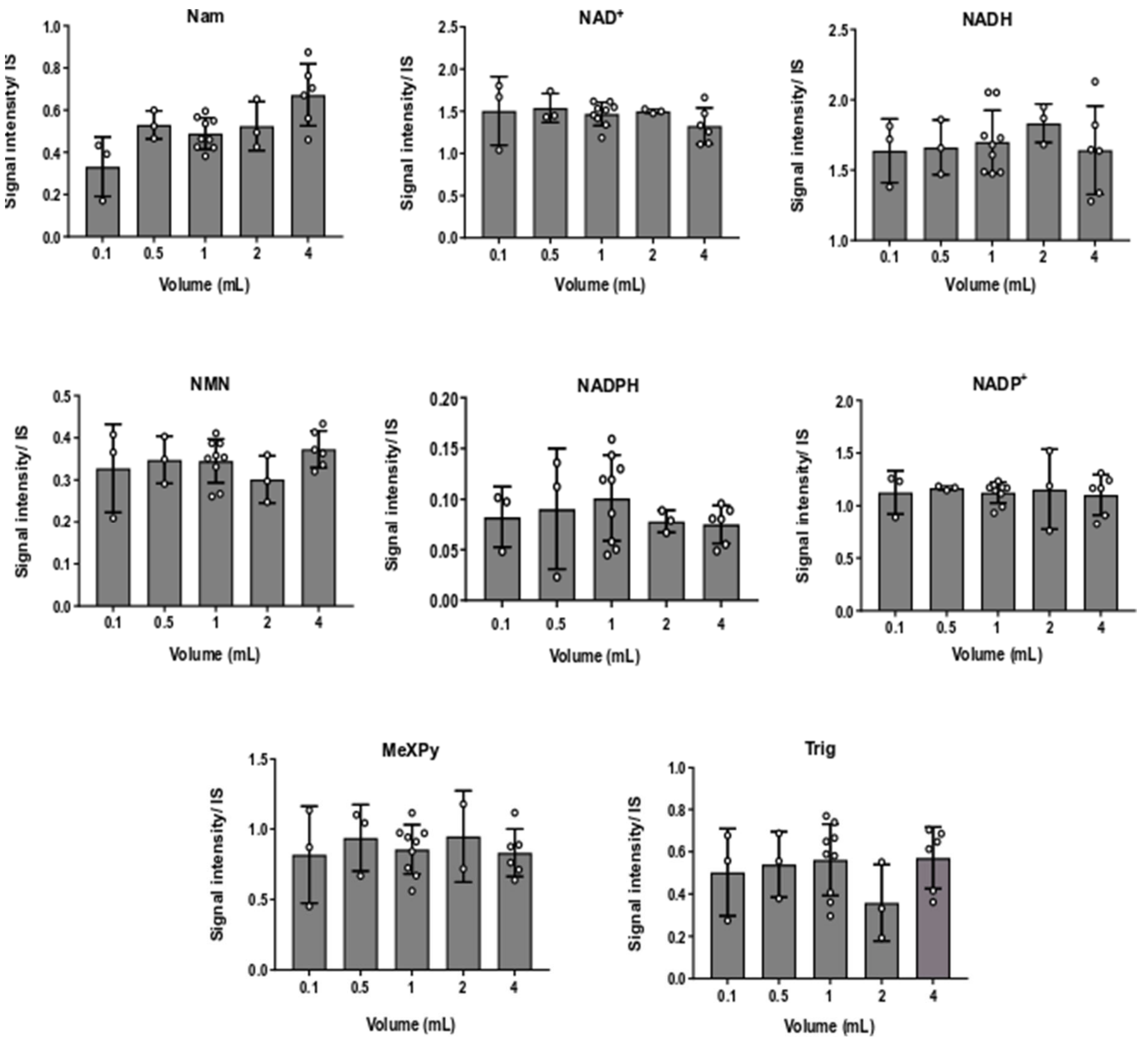

Figure S4. LC-MS metabolite signal intensity per internal standard for NAD$^+$ metabolites in human whole blood according to aliquot volume (0.1, 0.5, 1, 2, 4 mL) at blood draw. Average ±standard deviation values from 3 donors (except for MeXPY at 2 mL, 2 donors were analyzed; per aliquot and subject *n* = 1 for 0.1 mL, 0.5 mL, 2 mL, *n* = 2 for 4 mL, and *n* = 3 for 1 mL).

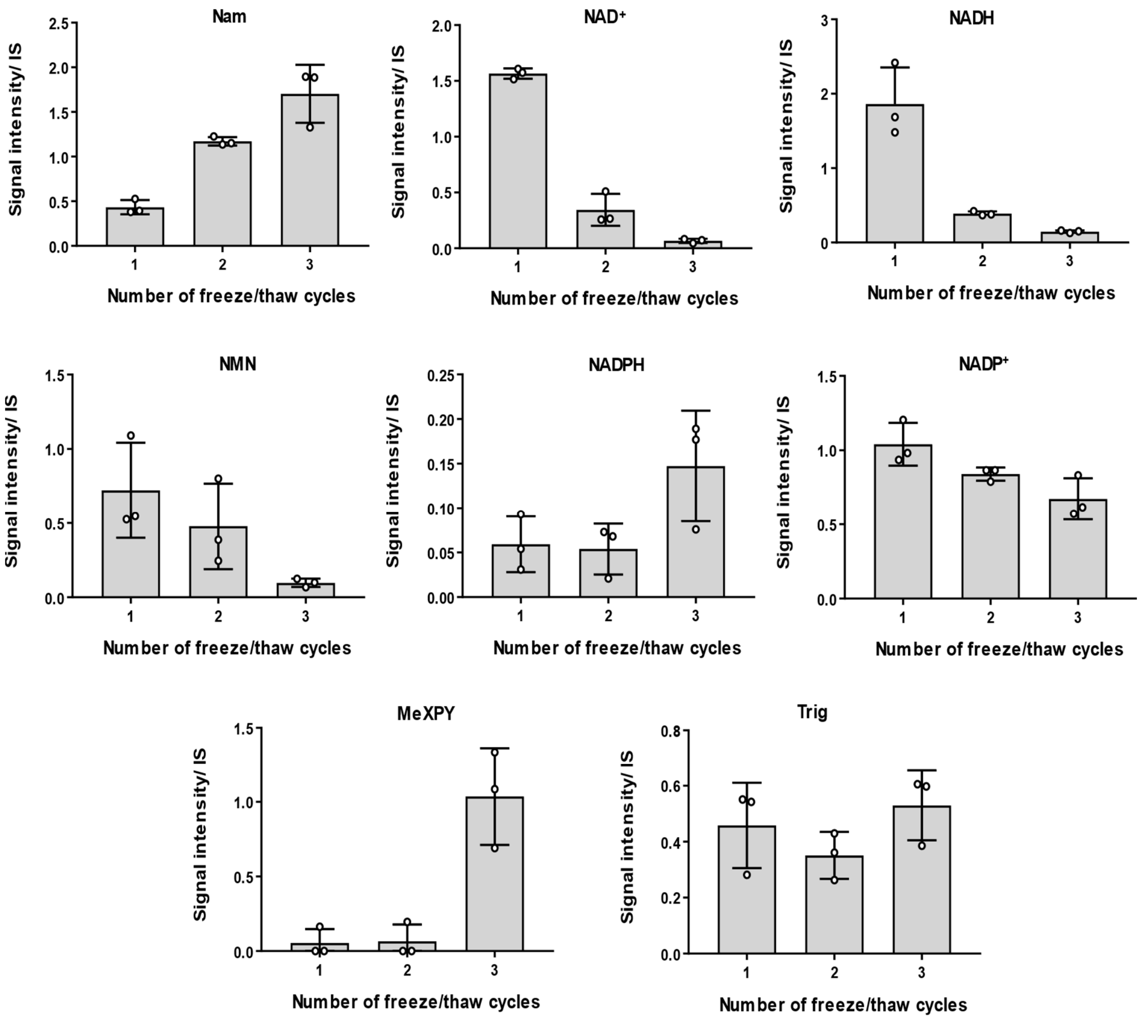

Figure S5. LC-MS metabolite signal intensity per internal standard for NAD^+ metabolites in human whole blood after 3 freeze-thaw cycles (1, 13, 21 days). Average ± standard deviation values from 3 donors (n = 3).

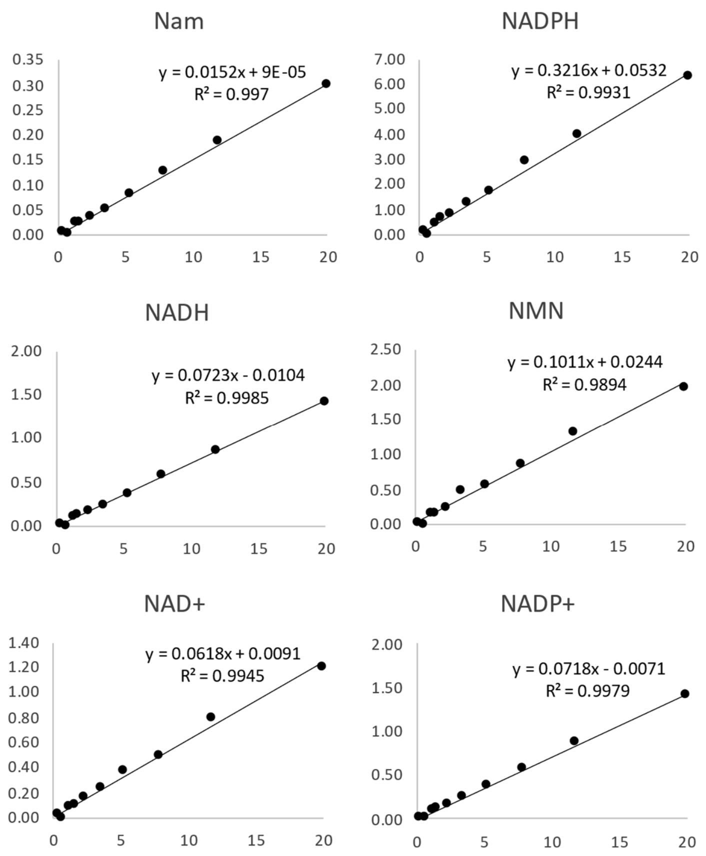

Figure S6. Calibration curves of NAD^+ metabolites relating LC-MS metabolite signal intensity per internal standard and metabolite concentrations (0.3-20 μM, 10 points). Average ± standard deviation values.

Table S4. NAD^+ metabolome quantification (expressed in μM) in human whole blood of 9 volunteers (average and standard deviation of 3 aliquots per donor).

**Average**	**Nam**	**NADH**	**NAD+**	**NADPH**	**NMN**	**NADP+**
D1	8.0	14.8	17.5	11.0	2.5	12.8
D2	7.8	14.7	17.7	16.9	2.2	15.8
D3	10.1	13.6	15.2	15.6	2.5	15.5
D4	8.8	15.8	18.1	15.3	2.5	19.3
D5	7.4	13.0	15.5	10.6	2.8	14.7
D6	9.8	13.4	15.6	11.1	2.0	14.2
D7	4.9	7.8	10.5	7.1	2.0	13.2
D8	7.9	13.2	15.6	8.8	3.5	19.0
D9	5.5	12.2	15.1	11.5	2.7	19.4
**St Dev**	**Nam**	**NADH**	**NAD+**	**NADPH**	**NMN**	**NADP+**
D1	0.3	1.4	2.0	0.3	0.4	1.2
D2	0.5	1.3	1.4	1.0	0.4	4.9
D3	1.4	1.2	1.6	2.5	0.6	5.7
D4	0.8	0.6	0.1	0.6	0.2	4.1
D5	0.7	1.3	1.1	1.2	0.1	4.3
D6	0.5	0.2	0.6	0.6	0.2	5.4
D7	0.3	1.0	1.4	0.8	0.0	2.7
D8	0.2	0.6	0.6	0.1	0.2	0.4
D9	0.4	1.0	0.4	0.4	0.3	5.9
Min	10.1	15.8	18.1	16.9	3.5	19.4
Max	4.9	7.8	10.5	7.1	2.0	12.8

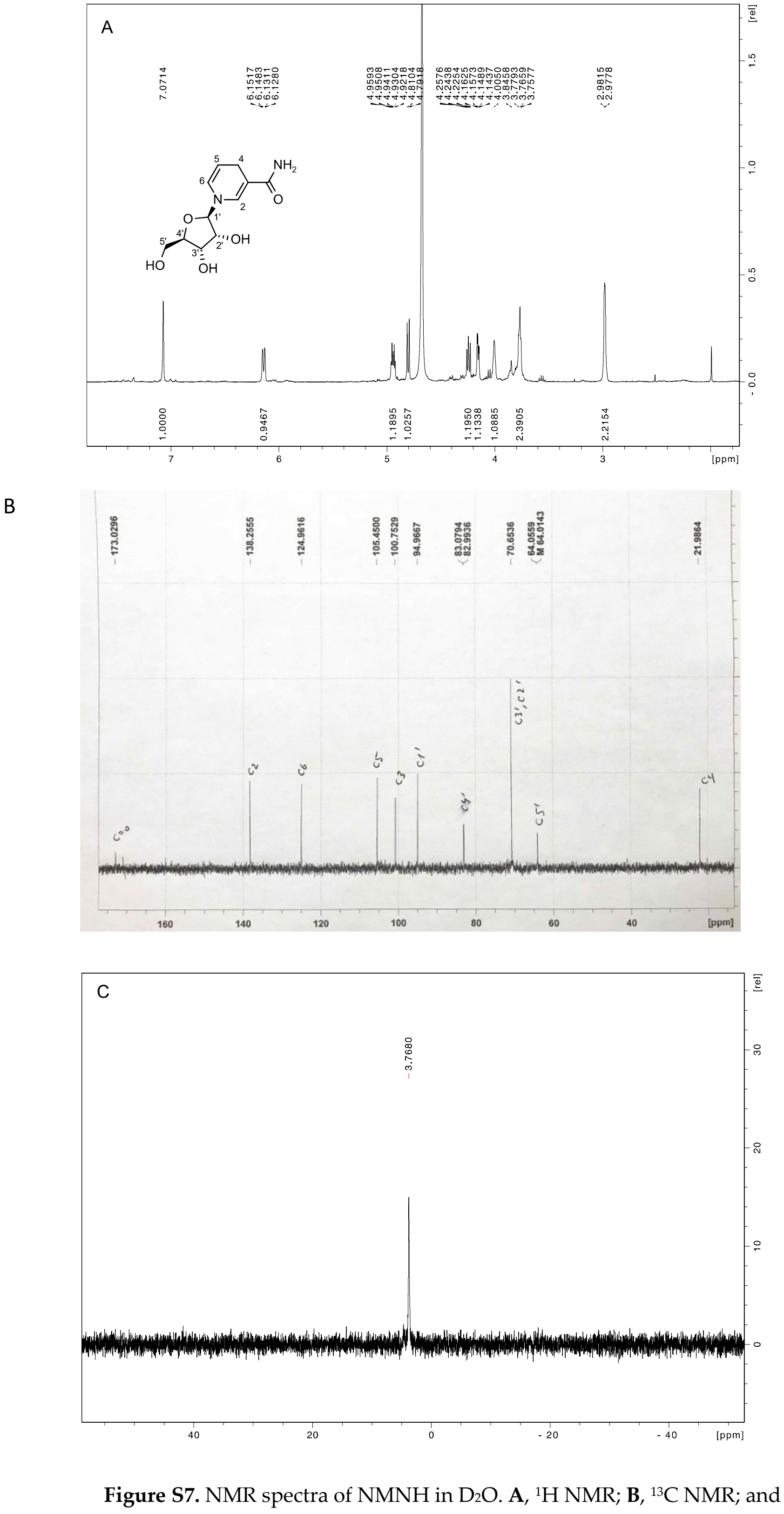

Figure S7. NMR spectra of NMNH in D_2O. **A**, ^{1}H NMR; **B**, ^{13}C NMR; and **C**, ^{31}P NMR.
